# Smoking habits and detection rate of unruptured intracranial aneurysms and incidence rate of subarachnoid haemorrhage in Norway between 2008 and 2015

**DOI:** 10.1007/s00701-020-04541-0

**Published:** 2020-08-28

**Authors:** Paulina Majewska, Sasha Gulati, Lise Øie, Øyvind Salvesen, Tomm B. Müller, Ole Solheim

**Affiliations:** 1 Department of Neurosurgery, St. Olav’s University Hospital, Trondheim, Norway; 2 Department of Neuromedicine and Movement Science, NTNU, Trondheim, Norway; 3 Department of Neurology, St. Olav’s University Hospital, Trondheim, Norway; 4 Unit for Applied Clinical Research, Faculty of Medicine, NTNU, Trondheim, Norway

**Keywords:** Subarachnoid haemorrhage, Unruptured intracranial aneurysm, Smoking, Incidence, Detection rate

## Abstract

**Objective:**

The aim of this study was to investigate the detection rate of unruptured intracranial aneurysms (UIAs) and incidence of aneurysmal subarachnoid haemorrhage (SAH) in relation to the rapidly changing smoking rates in Norway between 2008 and 2015.

**Methods:**

The registry-based study included all patients (≥ 16 years old) admitted to a hospital in Norway between 2008 and 2015 with a primary diagnosis of aneurysmal SAH or an outpatient diagnosis of UIAs. Age group–specific and total detection rate of UIAs and incidence rate of SAH over the years were calculated. Age group–specific data on smoking habits was retrieved from a national annual survey representative of the whole Norwegian population.

**Results:**

The rate of daily smokers decreased by 48% between 2008 and 2015. The detection rate of UIAs decreased by 47% from 17.3 in 2008 to 9.3 per 100,000 persons in 2015, and the incidence of SAH decreased by 30% from 11.3 in 2008 to 7.9 per 100,000 persons in 2015. The average annual decline in prevalence of daily smoking, UIA detection rate, and SAH incidence was 6.9%, 6.7%, and 4.3% per year, respectively. Multinomial logistic regression analyses revealed that the correlation between the decline in estimated daily smoking rates and decline in detection rate of UIAs (hazard ratio 52.5 CI = (14.9,∞), *p* < 0.00001) and incidence of SAH (hazard ratio 11.8 CI=(5.6,32.5), *p* < 0.00001) are statistically significant. The association is particularly strong in young and middle-aged cohorts (< 66 years old).

**Conclusion:**

It is likely that reducing cigarette smoking on a population-based level strongly reduces the rates of UIAs and SAH.

## Introduction

The estimated prevalence of intracranial aneurysms (IAs) is around 2% [1, 2], and the average annual risk of rupture is below 1%[3]. Many studies have found that smoking is one of the most important modifiable risk factors for both developing IA and subsequent rupture leading to subarachnoid haemorrhage (SAH) [4–7]. However, to what extent change in smoking habits in the general population affects the epidemiology of unruptured intracranial aneurysms (UIA) and aneurysmal SAH has not been much studied.

In this registry-based study, we investigate the detection rate of UIAs and incidence of aneurysmal SAH in relation to the rapidly changing smoking rates in Norway between 2008 and 2015.

## Methods

Study data have been collected retrospectively using records from the Norwegian Patient Registry (NPR), Norwegian Centre for Research Data (NSD), and Statistics Norway.

### UIA detection rate and incidence of SAH

All patients at the age of 16 or above admitted to hospital in Norway between 2008 and 2015 with either a primary diagnosis of aneurysmal SAH (ICD-10 codes I60.0-I60.7) or an outpatient diagnosis of UIAs (ICD-10 code I67.1) have been retrieved from the NPR. NPR is a national registry where information regarding diagnoses when patients receive inpatient or outpatient medical treatment by public specialist health-care services is automatically recorded. In Norway, acute illness requiring hospital admission is treated free of cost by the public health-care system. Only public hospitals provide treatment to patients with SAH [8]. Validity of NPR diagnoses has been investigated in a recent study which has shown that the positive predictive value for inpatient diagnosis of SAH is 95.3% [9]. Patients’ age at diagnosis was recorded. The age distribution of the Norwegian population for the time period was retrieved from Statistics Norway, the Norwegian national statistics bureau that prepares and publishes official statistics in Norway. The agency publishes yearly data on the Norwegian population including number of inhabitants and their age distribution [10]. Age group–specific and total detection rate of UIAs and incidence rate of SAH over the years were calculated. Due to anonymity requirements and strict privacy concerns, individuals below the age of 26 were excluded from the analysis of UIA detection rate due to very low detection rates.

### Smoking status

Age group–specific data on smoking habits among the Norwegian population for the same time period was retrieved from NSD, the Norwegian national archive for research data. The archive stores data from annual reports on smoking and travelling habits among the Norwegian population since 1973 [11]. The national annual survey includes participants representative of the whole Norwegian population. Between 2008 and 2015, each survey included on average 1182 participants. The percentage of current smokers in each age group during the years was calculated.

### Statistical analysis

Smoking proportions in each age group and year were estimated using logistic regression with age group as a factor and year as a covariate including the interaction between age group and year. The counts of events across years for each age group were assumed multinomially distributed conditional on the total count of events for that age group. By using estimated smoking proportions and population sizes for each year within an age group, the multinomial probabilities depend only on the hazard ratio (HR) due to smoking. The HR was then estimated using maximum likelihood. The HR was tested for significance using the Wilks likelihood ratio test. The confidence interval for the HR was found as the set of HRs not rejected by Wilks likelihood ratio test. The statistical analysis was performed using R version 2.13.1. Statistical significance level was set at *p* < 0.05.

### Ethics

Ethics committee approval was unnecessary for this study due to the fact that all data used were anonymous. This article does not contain any studies with human participants performed by any of the authors.

## Results

The rate of daily smokers decreased by 48% between 2008 and 2015. The average annual decrease in prevalence of daily smoking was 6.9% during the study period. As seen in Table 1, the largest decline was observed among the youngest Norwegian population between 16 and 35 years of age (64% decrease). The smallest change in smoking rates was observed among individuals at the age 66 or older (27% decline).

**Table 1:** Percentage of daily smokers in Norway between 2008 and 2015

Year	Average percentage of daily smokers per age group		
	16–35	36–65	66 or above
2008–2010	16.2	22.4	15.0
2011–2013	10.2	18.6	9.9
2014–2015	5.9	14.0	11.0

The detection rate of UIAs decreased by 47% from 17.3 in 2008 to 9.3 per 100,000 persons in 2015. The average annual decline in UIA detection rate was 6.7%. Decrease in UIA detection rate was observed in all age groups (≥ 26 years of age) apart from among individuals 76 years old and older.

The incidence of SAH decreased by 30% from 11.3 in 2008 to 7.9 per 100,000 persons in 2015. In the study period, the average annual decline in SAH incidence was 4.3% per year. Decline in SAH incidence rate was observed in all age groups (≥ 16 years of age). The largest decrease in SAH incidence rate was observed among individuals between 36 and 45 years of age (49% decline) while the smallest decrease was seen among individuals between 66 and 75 years of age (10.5% decline).

Multinomial logistic regression analysis revealed that there was a statistically significant correlation between the decline in estimated daily smoking rates and the decline in detection of UIAs (hazard ratio 52.5, 95% CI = (14.9,∞), *p* < 0.00001) and SAH incidence (hazard ratio 11.8, 95% CI = (5.6,32.6), *p* < 0.00001).

**Fig. 1:**
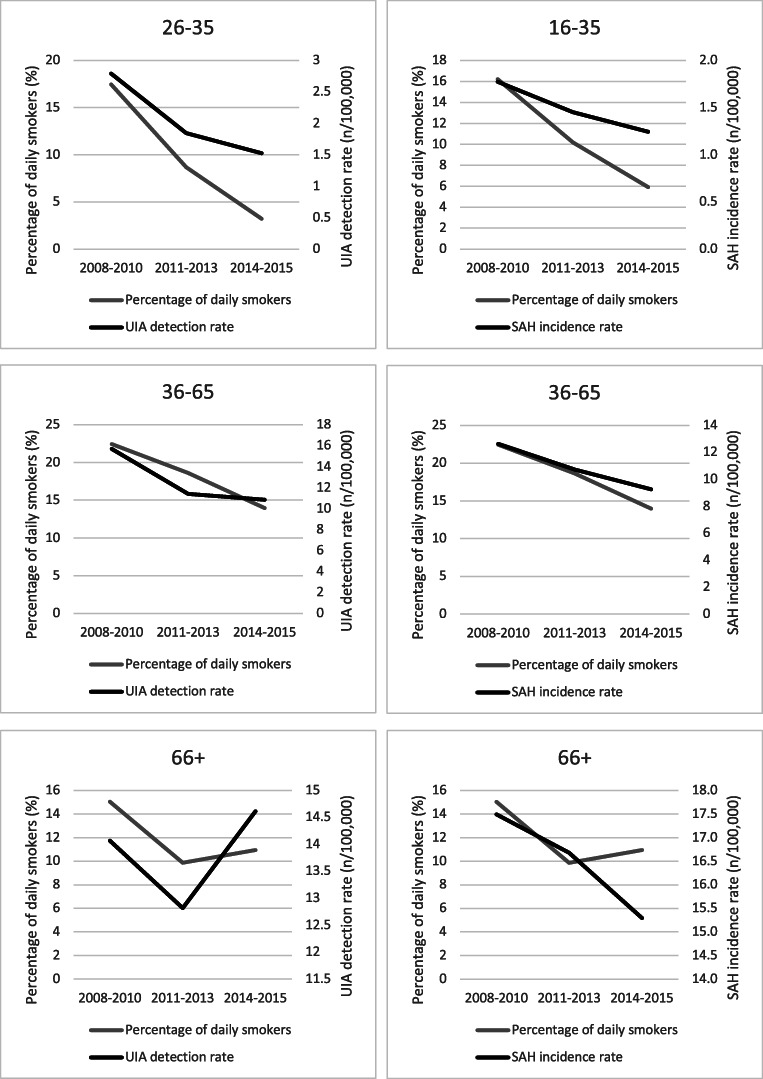
Association between detection rate of UIA and incidence rate of SAH and rate of daily smokers in different age groups in Norway between 2008 and 2015

As seen in Fig. 1, the association between the prevalence of daily smoking and the detection rate of UIA or incidence rate of SAH seems strongest among age groups younger than 66 years.

## Discussion

In the current study, we observed a large and gradual decrease in both the annual detection rate of UIAs and incidence rate of SAH in Norway between 2008 and 2015. In the study period, the annual decline in SAH incidence in Norway was 4.3% per year. The percentage of daily smokers in the population fell gradually at the same time. We found a strong correlation between the reduction in daily smoking and the decline in observed detection rate of UIAs and incidence rate of SAH in the same time period. The association was particularly strong in young and middle-aged cohorts (< 66 years old).

Our findings are in line with a recent study from Ireland that also observed an overall correlation between smoking rates and incidence rates of SAH in the Irish population [12]. Declining incidence rates of SAH were also reported in a systematic review published in 2007. The review included 42 studies performed worldwide, and reported a decrease in SAH incidence rates of 0.6% per year [13]. This trend has also been shown in previous epidemiological studies in Norway [14] and Finland [15], and the authors of the latter study speculated that the declining incidence of SAH was linked to the decreasing rate of daily smoking among the Finnish population [15]. Out of the mentioned studies, only the Irish and Finnish studies presented data on populational smoking habits. In the latter study, no statistical analysis investigating the correlation between the incidence of SAH and smoking was performed. Our study is the first one to report data on SAH incidence rate and smoking rates in different age cohorts.

The decline in SAH incidence has not been observed in all countries. A recent study from the USA found stable regional incidence rates of SAH between 1988 and 2010 [16]. The incidence of SAH has also been stable in Australia between 1998 and 2008 [17]. Moreover, a study from Hong Kong, China, has found an increase in incidence of SAH from 4.1 per 100,000 person-years in 2002 to 5.6 in 2010 [18]. An international report on smoking habits between 2006 and 2012 showed that the annual prevalence of daily smoking reduced by 4.1% per year in Norway and approximately 2% in Australia, Finland, and USA. All mentioned countries had similar prevalence of daily smokers in 2012 (between 15.8 and 17.9). At the same time, the annual prevalence of daily smoking increased by 0.2% per year in China and was as high as 24.2% in 2012 [19]. We believe that the national epidemiological trends of SAH incidence may partially be explained by the change in smoking habits in different countries.

In addition, our study presents a novel finding of declining detection rate of UIAs despite a gradual increase in the availability of MR imaging over time [20]. The decline was also significantly associated with smoking reduction. The association is likely to be genuine as many UIAs are incidental findings, and a likely increased use of sensitive diagnostic imaging in the study period would have counteracted and reduced the observed correlation with smoking.

According to our findings, the link between the change in smoking habits and aneurysm development and their rupture seems less obvious among the elderly. Although smoking is an important risk factor for both aneurysm formation and rupture, there are other risk factors that may play a relatively larger role in various age cohorts. We suspect that a cumulative effect of the risk factors is more likely to play an important role among the older population.

Our findings indicate that reducing cigarette smoking on a population-based level strongly reduces the rates of UIAs and SAH. The findings are important not only for clinicians who advise against cigarette smoking especially among patients with increased risk of IAs development and their rupture but also health-care policymakers and public health prevention agencies. In 2004, the Norwegian government introduced a policy guaranteeing the inhabitants of Norway “access to air without cigarette smoke,” according to which smoking at work or in public places is prohibited. Smoking rates subsequently fell [14]. However, not all countries have developed policies against cigarette smoking in public places especially in the developing world where the rates of subarachnoid haemorrhage are almost twice as high as in developed countries [4]. The observed large effect of change in smoking habits on SAH epidemiology should encourage policymakers in other countries to impose stricter smoking legislation.

The present retrospective registry-based study has its limitations. Although the positive predictive value for inpatient diagnosis of SAH is as high as 95.3% based on NPR data [9], the study does not include SAH cases with prehospital death. Furthermore, data on smoking habits have been collected as part of a survey investigating only a sample of the population and, hence estimated and not actual smoking rates were used in the data analysis. Nevertheless, it is a national survey depicting results representative of the whole country [21]. Moreover, we cannot exclude that other unmeasured factors like antihypertensive or lipid modifying drugs may have played a role in changing the epidemiology of UIAs and SAH. According to the Norwegian Prescription Database, a national registry that contains data on all dispensed drugs in Norway, the number of users of antihypertensive drugs and other drugs used for cardiovascular disorders increased by 4.9% per 100,000 inhabitants from 2008 to 2015, with an average annual percentage increase of 0.7% per year [22]. Still, changes in drug prescriptions over time are rather modest compared with the enormous change in smoking rates and SAH in the same period.

## Conclusions

The annual detection rate of UIAs and incidence rate of SAH, as well as the percentage of daily smokers decreased gradually in Norway between 2008 and 2015. Our study found a strong correlation between the reduction in daily smoking and the decline in detection rate of UIAs and incidence rate of SAH, especially in young and middle-aged population (< 66 years old). Public measures or legislation that affect smoking habits in the general population are likely to have a large impact on the epidemiology of IAs and SAH.

## Data Availability

The data that support the findings of this study are available on request from the Norwegian Patient Registry, Norwegian Centre for Research Data and Statistics Norway. The data are not publicly available due to privacy and ethical restrictions.
